# Bi^3+^-Related Multimode Emission in Garnet: A First-Principles Study

**DOI:** 10.3390/ma18225082

**Published:** 2025-11-08

**Authors:** Bin Jiang, Qing Liu, Fengfeng Chi, Bibo Lou

**Affiliations:** 1 College of Electronic and Information Engineering, West Anhui University, Lu’an 237012, China; qianyue@wxc.edu.cn; 2 New Energy Technology Engineering Laboratory of Jiangsu Province, School of Science, Nanjing University of Posts and Telecommunications, Nanjing 210023, China; ffchi@njupt.edu.cn; 3 School of Integrated Circuits, Chongqing University of Posts and Telecommunications, Chongqing 400065, China; loubb@cqupt.edu.cn

**Keywords:** first-principles, Bi^3+^ and Bi^3+^ dimer, multiband emission, transition process

## Abstract

In this work, systematic first-principles calculations were performed to investigate the multiband emissions of Bi-doped Y_3_Ga(Al)_5_O_12_ phosphors. The predicted emissions of Bi^3+^ show that the violet narrow-band emission can be attributed to the ^3^P_1_–^1^S_0_ transition of Bi^3+^ at Y sites, and both the metal-to-metal charge transfer (MMCT) of Bi^3+^ at Ga (Al) sites and the luminescence of Bi^3+^ dimers can generate visible emissions. Detailed formation energy calculations subsequently rule out the possibility that the visible emission originates from the MMCT of Bi^3+^ at Ga (Al), as the concentration of Bi_Y_ is much greater than that of B_Ga_ (or Bi_Al_). To better understand the relationship between the nephelauxetic effect and the coordination environment, the vacuum-referred binding energy (VRBE) model was utilized to determine the energy levels of bismuth ions relative to the vacuum level in different systems and at different sites. The results provide insight into the relationship between the coordination environment and the emission properties of Bi^3+^ and are helpful for analyzing and optimizing the luminescent properties of bismuth-doped garnet-like materials.

## 1. Introduction

Garnets, a family of cubic-phase crystal structures, have attracted considerable interest as host materials for phosphors [1]. The unique structural rigidity [2] of garnets provides an excellent platform for activators to achieve exceptional luminescence, e.g., Cr^3+^-[3,4], Nd^3+^-[5,6], Eu^3+^-[7,8], and Ce^3+^-doped garnet [9,10]. Among these, Bi^3+^-doped Y_3_Al_5_O_12_ (YAG) and Y_3_Ga_5_O_12_ (YGG) exhibit promising multifunctional luminescent properties due to their highly environment-sensitive electron structures [11,12,13]. Under violet excitation, YAG:Bi (YGG:Bi) typically exhibits multiband emission, with emission ranging from violet to visible light. Its violet emission shows potential for applications in photochemistry and phototherapy [14] due to its short lifetime and long afterglow properties. Meanwhile, the multiband and wide full-width at half-maximum (FWHM) visible emission offers potential applications in temperature sensing [15], LEDs [16,17,18], and anti-counterfeiting [19]. Clarifying the origin of this complex emission is essential for enhancing the optical properties and designing novel materials. Previous studies have focused on the experimental emission phenomenon, and empirical analyses of emission origins have led to inconsistent conclusions. For instance, both the visible and violet emission have been related to the ^3^P_1_–^1^S_0_ transition of Bi^3+^ at Y sites [20,21,22].

Generally, trivalent bismuth ions possess a 6s^2^ ground state and an environment-sensitive 6s6p excited state. In specific host materials, Bi^3+^ can accept an electron from the valence band through charge transfer (CT) excitation or release a 6s electron to the conduction band via metal-to-metal charge transfer (MMCT) [11,12,13]. These excited states can generate emissions ranging from violet to visible, posing a challenge in distinguishing the origin of emissions. Due to the lack of detailed electron structure information of bismuth ions, it is difficult to distinguish the emission of Bi^3+^ solely from experimental spectra. A systematic theoretical investigation can provide insights into electron structures to transition processes, thereby enabling a rational understanding for designing novel materials.

In the present work, density function theory (DFT) calculations were performed on Bi^3+^-doped garnets Y_3_(Ga/Al)_5_O_12_ to explore the properties of bismuth ions. First, the formation energies of bismuth-related defects and Schottky-type intrinsic defects were calculated. Then, the site occupation of bismuth was clarified. Secondly, the electron structure of bismuth at Y sites, as well as at Ga (Al) sites, was investigated to identify the relationship between the nephelauxetic effect, crystal field splitting, and the coordination environments. Then, the VRBE model was utilized to clarify the energy level of bismuth at the band edge. Lastly, both the optical properties of single bismuth ions and their dimer were calculated, based on the constrained DFT and Δ self-consistent field method. For the single Bi^3+^, the lowest excited state was identified, and the corresponding excitation and emission processes were checked. For the Bi^3+^ dimers, the bonding type and the excitation and emission processes were discussed. Moreover, the mechanics of afterglow luminescence observed in YAG:Bi were briefly discussed.

## 2. Computation Details

All calculations were carried out within the density function theory framework, as implemented in the Vienna Ab initio Simulation Package (VASP) [23,24]. The initial host structures were obtained from the Inorganic Crystal Structure Database [25], and the PBEsol exchange–correlation function was employed for structure optimization [26]. The interactions between the core and the electrons were described by the PAW methods, with a plane-wave cutoff energy of 500 eV. Y(4s^2^4p^6^5s^1^4d^2^), Al(3s^2^3p^1^), Ga(3d^10^4s^2^4p^1^), O(2s^2^2p^4^), and Bi(5d^10^6s^2^6p^3^) were treated as the valence electrons. Since the crystal structures of YAG and YGG obtained from the database belong to the Ia$\bar{3}$d space group, which contains 160 atoms and is beyond the computational capabilities of our limited source, the primitive cells of YAG and YGG were adopted for the relaxation of pristine and doped structures. A 5 × 5 × 5 k mesh was used for sampling the Brillouin zone of the pristine cells, while only the single Γ point was employed for optimizing defective structures. The atoms were fully relaxed until the Hellmann−Feynman forces on all atoms were less than 0.02 eV/Å. Spin–orbit coupling (SOC) was included to optimize the configuration of Bi^2+^ and the excited state of Bi^3+^. To overcome the underestimation of the band gap in the PBEsol calculation, the hybrid functions HSE06 [27] and PBE0 were employed. For the PBE0 function, the Hartree–Fock exchange fraction α (30% for YAG and 27% for YGG) was set as 1/ε_∞_ [28], where ε_∞_ is the dependent dielectric constant, as listed in Table 1. In order to conserve limited computing resources, a single Γ point was employed in all hybrid DFT calculations. Given that YAG and YGG possess identical space groups, the high symmetry k-points along the path Γ-H-N-Γ-P-H and P-N in the Brillouin zone were employed to calculate the band structure of YAG and YGG [29].

For Bi-doped systems, the Δ self-consistent field (ΔSCF) method [30] was employed to calculate the energies of Bi-related excited states. The ground state ^1^S_0_ of Bi^3+^ is represented as two paired 6s electrons: one occupying the majority (spin-up) 6s orbital and the other occupying the minority (spin-down) 6s orbital. The excited state ^3^P_1_ noted as the 6s6p configuration of Bi^3+^ is modeled as a spin minority (spin-down) 6s electron of Bi^3+^ moving to the spin majority (spin-up) 6p orbital of Bi^3+,^ with the other 6s electron remaining unchanged. Since the valence band edge is primarily composed of the 2p orbitals of oxygen elements, the configuration of the charge transfer (CT) state is presented as Bi^2+^ bound to a hole at the valence band edge. Similarly, the excited state configuration of the metal-to-metal charge transfer (MMCT) state is represented as Bi^4+^ bound to an electron at the CBM.

Generally, the formation energy of a defect system with charge q is defined as [31]

(1) $$E^{f}\left(X^{q},E_{F}\right)=E_{\mathrm{tot}}\left[X^{q}\right]\;-{\;E}_{\mathrm{tot}}\left[\mathrm{bulk}\right]\;-\sum_{i}n_{i}\mu_{i}+qE_{F}+E_{\mathrm{corr}}$$

where E_tot_[X^q^] is the total energy of the defective system, E_tot_[bulk] is the total energy of the pristine cell, n_i_ is the variation in the atoms, and μ_i_ is the chemical potential of the corresponding atom. E_F_ is the electron chemical potential released as the Fermi energy. E_corr_ is the correction term of electrostatic interactions resulting from image charges of charged cells. Thus, the thermodynamic charge transition level (CTL), which is noted as the formation energy difference between q_1_ state and q_2_ state in the equilibrium configuration of each charge state, is defined as

(2) $$\varepsilon \left(q_{1}/q_{2}\right)=\frac{E^{f}\left(X^{q_{1}};E_{F}=0\right)-E^{f}\left(X^{q_{2}};E_{F}=0\right)}{q_{2}-q_{1}}$$

In practice, the transition process, including excitation and emission, takes place within a geometric configuration that remains unchanged, and then the thermodynamic charge transition level is recorded to the zero-phonon line, based on the Franck–Condon principle.

For the charged cells, the finite size of the cell gives rise to electrostatic interactions between periodic images of the cell. Here, the Lany–Zunger (LZ) correction [32] is chosen and denoted as

(3) $$E_{\mathrm{corr}}=\left(1+f\right)\frac{q^{2}\alpha_{M}}{2\varepsilon L}$$

where q is the charge state of the cells; α_M_ is the Madelung constant and is approximated to be 2.837; the value of (1 + f) is assumed to be 2/3; ε is the dielectric constant of the materials; and L is the separation between defects and is denoted as the cubic root of cell volume. The calculated correction values are listed in Table 1.

**Table 1:** The experimental dielectric constant and the finite-size effect correction (in eV) for the charged cell.

	ε_∞_	ε_0_	L (Å)	E_corr_(∞)	E_corr_(0)
YAG	3.28 [33]	12.01 [34]	10.39	0.40	0.11
YGG	3.72 [34]	12.60 [34]	10.63	0.34	0.10

## 3. Results

**Figure 1:**
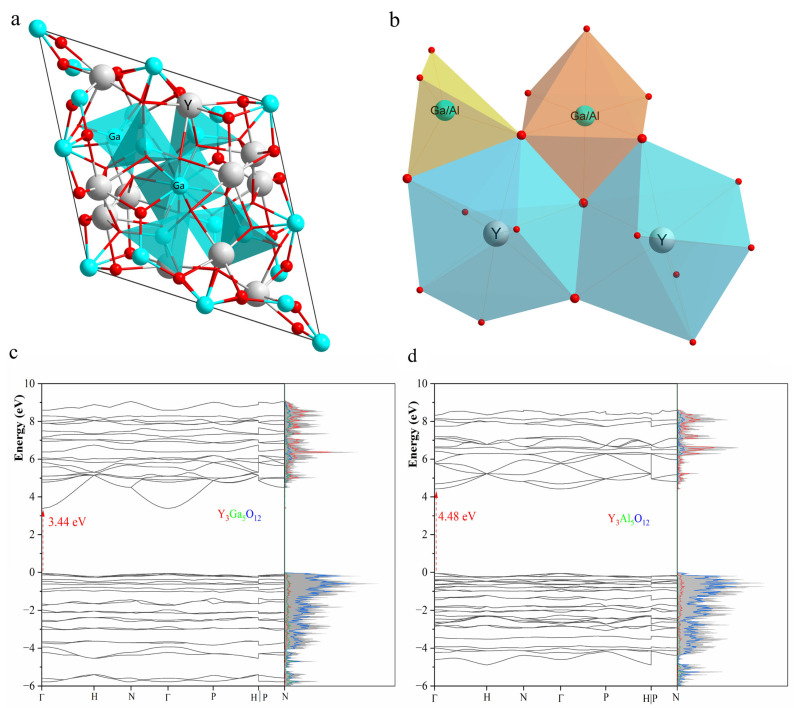
Crystalline structures of Y_3_Ga_5_(Al_5_)O_12_ (**a**) consisting of [YO_8_] polyhedron and edge-sharing clusters (**b**). Band structure and density of states of (**c**) YGG and (**d**) YGG calculated by the PBEsol function.

As shown in Figure 1a, the converted YGG and YAG cells each contain 12 Y atoms and 20 Ga and or Al atoms. The Bi-doping concentrations are 8% for Y sites and 5% for Ga (Al) sites, consistent with the experimental values. The lattice parameters of unrelaxed converted cells taken from the cubic crystal are 10.396 Å for YAG and 10.692 Å for YGG. After structural optimization, the inherent deficiencies of the PBEsol functional induce a lattice parameter shrinkage of 0.1% for YAG and 0.6% for YGG, as listed in Table 2.

The Y sites exhibit a distorted [YO_8_] polyhedron (D_2_ point group) and share edges with the GaO_4_ (S_4_ point group) and GaO_6_ (S_6_ point group) clusters, forming a Y-Ga-Y framework, as shown in Figure 1b. The GaO_4_ cluster, which exhibits a distorted tetrahedron, performs corner sharing with the distorted octahedron GaO_6_ cluster. Although the Y^3+^ and Ga^3+^ (Al^3+^) are both trivalent, Bi^3+^ tends to substitute Y sites rather than Ga or Al sites because of their similar ionic radius [35]. However, previous studies have reported emissions from Bi^3+^ ions doped with ionic radius-mismatched cations, such as those in Bi^3+^-doped ZnGa_2_O_4_ and Zn_2_GeO_4_ [36,37]. Therefore, Bi^3+^ substituting Ga^3+^ and Al sites are also calculated.

**Figure A2:**
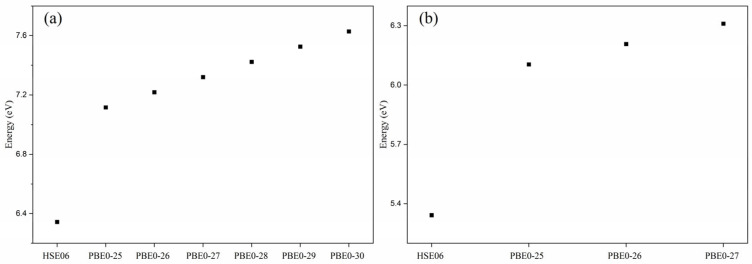
The influence of Hartree–Fock exchange fraction α in PBE0 on the band gap of (**a**) YAG and (**b**) YGG. Here, PBE0-α refers to the PBE0 functional with a Hartree–Fock exchange fraction of α.

Figure 1c,d show the band structure and density of states (DOSs) of YAG and YGG, calculated with respect to the optimized geometries. Both YAG and YGG exhibit a direct band gap within the PBEsol function, with the conduction band maximum (CBM) and valence band minimum (VBM) located at the Γ point. The PBEsol-calculated band gaps are 3.44 eV for YGG and 4.48 eV for YAG, which are significantly underestimated compared to the experimental values of 5.50–6.30 eV for YGG and 6.00–7.00 eV for YAG [38,39,40]. In contrast, the HSE06 function yields results matching the experimental results well, as shown in Figure A2. As depicted in Figure 1c,d, the valence band edge in both YAG and YGG is mainly composed of the 2p states of oxygen ions, and the conduction band edge is dominated by the 5s states of Y ions and the 3p states of Ga or Al ions.

**Table 2:** The PBEsol optimized and experimental parameters (Å) of YAG and YGG.

	Calculated			Experimental [41,42]		
	a	α	volume	a	α	volume
YAG	10.385	109.471	862.203	10.396 [41]	109.471	864.951
YGG	10.631	109.471	924.878	10.692 [42]	109.471	940.797

**Figure 2:**
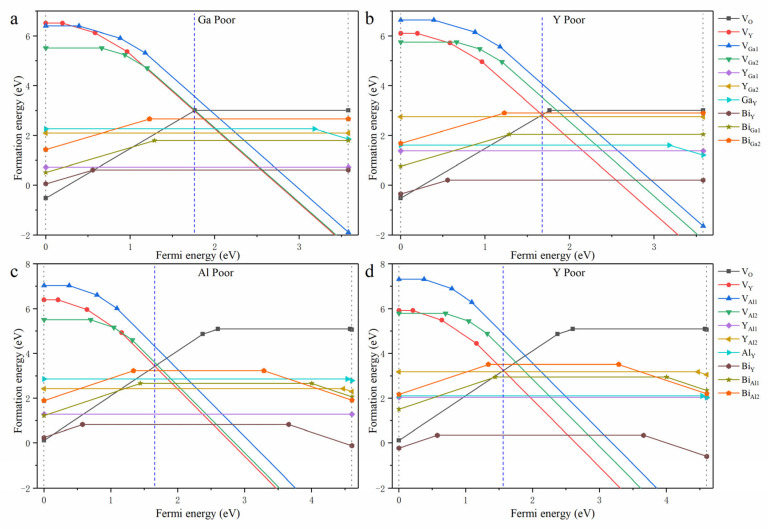
The formation energies of intrinsic defects and bismuth-related defects in YGG (**a**,**b**) and YAG (**c**,**d**). The grey dot lines in the figure are the VBM (**left**) and CBM (**right**), and the blue dashed lines are the Fermi level determined by the neutral condition. All results were obtained using the PBEsol function.

As indicated by Equation (2), the formation energy of a defect is related to the atomic chemical potential, which can be determined by the thermodynamic constraints that correspond to the actual synthesis process. It is found that the Gibbs free energy, which is equal to the chemical potential, can be estimated by the total energy calculated in DFT. The chemical potential of a compound can be represented as the sum of the chemical potential of each constituent element. Accordingly, the chemical potentials of Y, Ga, Al, and O are subject to the following relation:

(4) $$3\mu_{Y}+5\mu_{A}+12\mu_{O}=\mu_{Y_{3}A_{5}O_{12}}$$

(5) $$\mu_{O}=\frac{1}{2}\mu_{O_{2}}+{\Delta \mu }_{O_{2}}$$

(6) $$2\mu_{Y}+3\mu_{O}=\mu_{Y_{2}O_{3}}+{\Delta \mu }_{Y_{2}O_{3}}$$

(7) $${2\mu }_{A}+{3\mu }_{O}=\mu_{A_{2}O_{3}}+{\Delta \mu }_{A_{2}O_{3}}$$

where A presents the Al and Ga elements, and Δμ is the chemical potential difference of Y_2_O_3_ and A_2_O_3_ in the formation of Y_3_A_5_O_12_ in an atmosphere where the oxygen has an excess chemical potential. In the experiments, the materials are synthesized in an air atmosphere at 1500 K with an oxygen partial pressure of 0.2 atm, where the oxygen-related Δμ is set to −1.12 eV, as reported by Lou et al. [13]. Here, Δμ is calculated under two extreme conditions: Y poor condition and Ga (Al) poor condition. The defect formation energy is then obtained and shown in Figure 2a–d.

At the Fermi level, determined as the cross point of the lowest positive and negative defect formation energy curves, bismuth ions at both Y sites and Ga(Al) sites exhibit a trivalent valence state. In both YGG and YAG, bismuth ions prefer to substitute Y sites owing to the fact that the Bi_Y_ defect has the lowest defect formation energy. The energy difference between Bi_Y_ and Bi_Ga_ under Ga poor conditions is 1.28 eV, which is slightly smaller than the corresponding difference between Bi_Y_ and Bi_Al_ under Al poor conditions. Under thermodynamic equilibrium, the concentration of defects can be determined by $N_{\mathrm{site}}\exp(-E^{f}/\mathrm{kT})$ [13]. YAG and YGG samples are synthesized at around 1500 K, corresponding to a kT of about 0.13 eV. Thus, the concentration of Bi_Ga1_ is around four in ten thousand of Bi_Y_ under Ga poor conditions and is reduced to one in a hundred thousand under Y poor conditions. In contrast, the corresponding concentrations of Bi_Ga2_ and Bi_Al_ are well below that for Bi_Ga1_. Since the concentration of bismuth doped in the materials ranges from 1% to 10%, the concentrations of bismuth at Ga sites and Al sites are estimated to be less than 0.001%. Consequently, the emission of Bi^3+^ at these sites should be greatly weaker than that of Bi^3+^ at Y sites.

**Figure A4:**
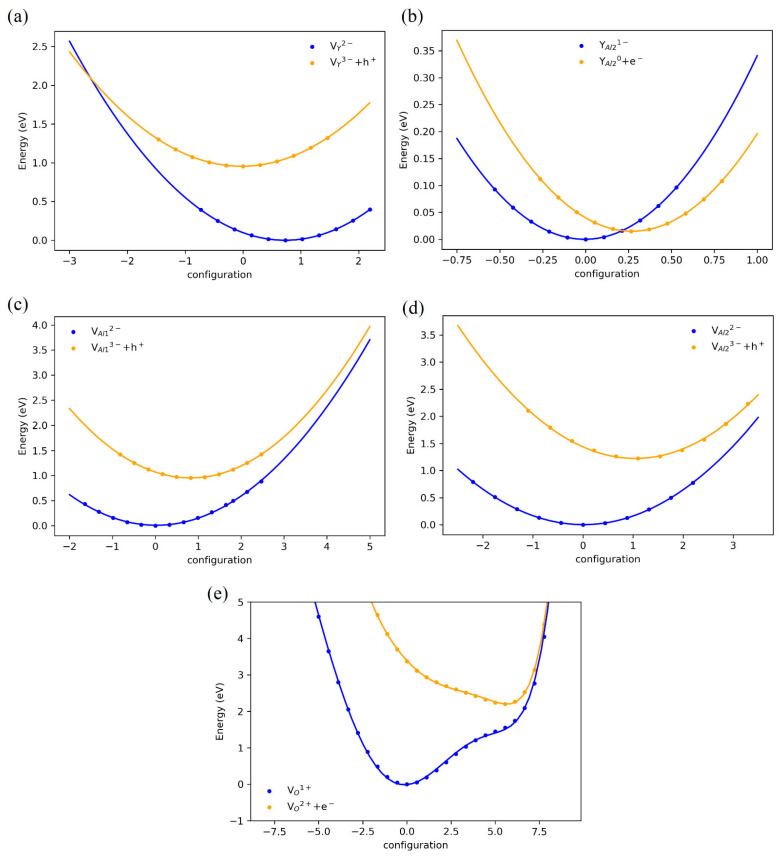
The coordination configuration of (**a**) V_Y_^3−^, (**b**) Y_Al2_^0^, (**c**) V_Al1_^3−^, (**d**) V_Al2_^3−^, and (**e**) V_O_^2+^ in YAG host. All results were obtained using the PBEsol function.

In addition, the formation energies of intrinsic defects were calculated to analyze the long afterglow of Bi^3+^ reported by Liu et al. [20]. Near the Fermi level, the dominant defects include Y_Al_ (Y_Ga_), Al_Y_ (Ga_Y_), V_O_, V_Y_, and V_Ga_ (V_Al_). Under Ga or Al poor conditions, Y_Ga_ and Y_Al_ defects form easily, whereas under Y poor conditions, Ga_Y_ or Al_Y_ defects are easily formed. For the Al_Y_ and Y_Ga_ anti-sites, the most stable states are neutral, exhibiting no potential as carrier traps. In contrast, Ga_Y_ and Y_Al2_ anti-sites possess charge state transition levels (CTLs) below the CBM, indicating their ability as electron traps. For oxygen vacancy, the dominant charge state is +2, and the associated CTL suggests a potential as a deeper electron trap. For Y and Ga (Al) vacancies, the dominant charge state is −3, suggesting their capability as deep hole traps. As an example, the energy barriers for carrier trapping by main defects in YAG are presented in Figure A4. These energy barriers are similar in magnitude to the corresponding CTLs. Compared to the thermoluminescence spectra measured by Liu et al. [20], the energy barriers of these vacancy defects are comparable to the experimental trap depths.

In experiments, Bi^3+^-doped YGG and YAG show multiband emissions in the violet to visible region. As listed in Table 3, the emissions of YGG:Bi^3+^ are located at 3.96 eV (violet region), 3.32 eV, 2.92 eV, 2.61 eV, and 2.53 eV (visible region). Similarly, the emissions of YAG:Bi^3+^ are located at 4.07 eV (violet region), 2.95 eV, and 2.63 eV (visible region). In comparison with the emission spectra of undoped YGG and YAG, the reported multiband emissions can be conclusively related to the bismuth ions. Based on this assignment, the optical properties of Bi^3+^ at Y sites and Ga(Al) sites are calculated, even though the Bi^3+^ is barely occupied at the latter.

**Figure 3:**
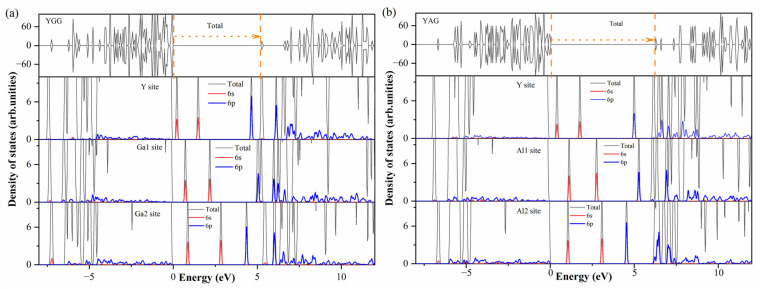
The DOS of Bi^3+^ calculated in the hybrid + SOC for YGG (**a**) and YAG (**b**), using the DOS of the primitive cell as a reference. The 6s and 6p orbitals of Bi^3+^ are represented by red and blue lines.

Figure 3a,b show the DOS of the 6s6p electron configuration of Bi^3+^. In our calculation, the ^3^P_1_ state of the Bi^3+^ ion is characterized by a spin-down 6s electron transfer to a spin-up 6p state with a hole trapped in the 6s state. As shown in Figure 3a, the unoccupied 6s orbital lies in the gap, and the occupied 6p orbitals are below the CBM with SOC included. When Bi^3+^ moves from Y sites to Ga1 sites, the occupied 6s and 6p orbitals migrate to the conduction band edge with the coordination number and average bond length of Bi-O decreasing, as listed in Table 4. Meanwhile, the splitting between 6p orbitals decreases as the point group changes from D_2_ to S_6_. A similar shift in the 6s orbital is obtained when the Bi^3+^ ion changes from Ga1 sites to Ga2 sites, with the coordination number and average bond length of Bi-O falling again. However, the occupied 6p orbital moves from the conduction band edge into the gap, associated with the reduction in the point symmetry. Similar trends are also observed in YAG, as shown in Figure 3b.

**Figure 4:**
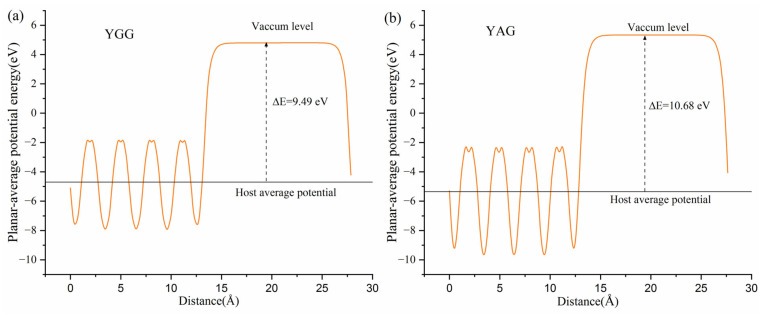
The vacuum level of YGG (**a**) and YAG (**b**) calculated by the PBE function.

To further understand the influence of the coordination environment, the vacuum-referred binding energies (VRBE) [43] model is adopted to explain the nephelauxetic effect. The VRBE model has previously been used to analyze coordination–environment-related effects in the RPO_4_ (R = Y, Lu, La) and RAlO_4_ (R = La, Y, Lu) systems [11,13]. Figure 4a,b show the planar average potential of YGG and YAG, as calculated by the slab method. The surfaces of YGG and YAG were modeled along the [001] orientation with a 20 Å vacuum layer along the c-axis orientation. The host average potential is set as the average potential across the garnet systems, while the vacuum level corresponds to the potential of the vacuum regions. The energy difference between the host average potential and the vacuum level is 9.49 eV for YGG and 10.48 eV for YAG. Unlike the RPO_4_ and RAlO_4_ systems, where only small energy variations are obtained upon substitution at R sites, a large energy difference is observed here when Ga is replaced by Al. This discrepancy is related to the significant variation in electronegativity between Ga and Al, which results in a substantial change in the nephelauxetic effect.

**Figure 5:**
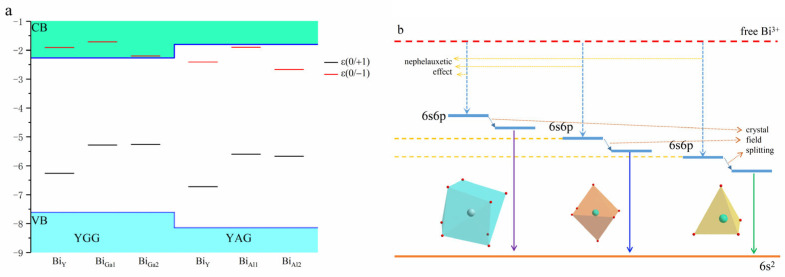
(**a**) VRBE mode of YGG and YAG with the band aligned to the vacuum level. (**b**) The sketch of nephelauxetic effect and crystal field splitting in three sites.

Figure 5a shows the VRBE model of YGG and YAG, with the band aligned to the vacuum level. The VBM of YGG is located at −7.69 eV, while the VBM of YAG is at −8.14 eV. In addition to the VBM and CBM, the transition levels of Bi^3+^ are also aligned with the vacuum level. All ε(0/−1) of Bi^3+^-doped YGG are inside the conduction band, indicating the instability of Bi^2+^ in YGG. However, the stable Bi^2+^ can be formed in YAG due to its larger band gap. Yet, the corresponding excitation and emission will also be large. When the Bi^3+^ ion changes from Y sites to Ga sites or Al sites, the ε(0/+1) shifts upward, while the ε(0/−1) shifts downward, with a notable exception: Bi_Ga1_ (Bi_Al1_) shows the highest transition level. As mentioned above, the Ga1 (Al1) sites present the highest point symmetry. This implies that variations in bond length and coordination number significantly influence the 6s and 6p orbitals. It is worth noting that the shifts in ε(0/+1) and ε(0/−1) follow a similar trend to the energy movements of the 6s and 6p orbitals, as shown in Figure 3. In summary, the nephelauxetic effect and crystal field splitting are illustrated in Figure 5b. As Bi^3+^ changes from the Y sites to the octahedron and tetrahedron sites, the reduction in bond length, coordination number, and the corresponding enhancement of nephelauxetic effect decrease the energy difference between the 6s6p state and the 6s^2^ state, resulting in a red shift from the violet to the visible range.

As listed in Table 3, the excitation energies of the so-called A band ^3^P_1_→^1^S_0_ transition are 4.35 eV for Bi_Y_ in YGG and 4.41 eV for Bi_Y_ in YAG. The corresponding emissions centers at 4.08 eV and 4.22 eV are in good agreement with the experiments. Meanwhile, for Bi^3+^ at Ga sites or Al sites, the excitation and emission of the A band undergo a red shift, due to the nephelauxetic effect and crystal field splitting. Except for the internal transition, ions with ns^2^ electron configuration, such as Bi^3+^, Sb^3+^, Pb^2+^, and Tl^4+,^ also exhibit so-called charge transfer transition [44,45,46,47]. For the MMCT, Bi_Y_ shows excitation energies of 4.32 eV in YGG and 5.24 eV in YAG, with the associated emission centers at 3.63 eV and 4.58 eV, respectively, resulting in a larger Stokes shift. For the MMCT of Bi^3+^ ions in Ga sites or Al sites, the excitation and emission exhibit a red shift of approximately 1.00 eV, compared to Bi_Y_. Due to their higher CTLs, the transition process of CT is not considered further and is excluded from Table 3. Thus, the violet emission can be assigned to the ^3^P_1_→^1^S_0_ transition, consistent with experimental assignments. Due to the reduced concentration of bismuth-doped Al sites, we tentatively relate the visible emission to Bi_Al_^3+^. However, for Bi^3+^ at Ga sites, a concentration as low as 0.01% can yield observable emission. For example, the effective concentration of Cr^3+^ is 0.01% to 0.1%. Moreover, the emission from bismuth ion-doped small-radius ions has been reported in ZnGa_2_O_4_ and Zn_2_GeO_4_ [36,37]. Thus, the 3.32 eV emission in YGG:Bi^3+^ may potentially originate from the A band of Bi_Ga_.

The emission performance of Bi^3+^ dimers has been investigated in several materials [13,48]. Its ground state is realized as [6s^2^,6s^2^], composed of two spin-up 6s electrons and two spin-down 6s electrons. Generally, the excited states of Bi^3+^ dimers can be classified into two types: the excited state ES1 is denoted as [6s3,6p1], where a 6s electron shared by two Bi^3+^ ions is excited to the hybrid 6p orbital; the other excited state, ES2, is realized as an inter valence charge transfer state [6s1,6s26p1], where a 6s electron from the first Bi^3+^ transfers to the 6p orbital of the second Bi^3+^. In general, the ES1 state can be detected in the system with inverse symmetry, and the ES2 state can be observed under all symmetry conditions.

For YGG and YAG systems, the bismuth ions mainly occupy Y sites. Therefore, only Bi_Y_^3+^-Bi_Y_^3+^ dimers are considered in this work. As listed in Table 5, the bond length between two Y sites is 3.8 Å and shrinks to 3.6 Å after Bi^3+^ substitution. The binding energy of dimers is calculated as E_binding_ = *E^f^*[Bi_A_^3+^ − Bi_B_^3+^] − {*E^f^*[Bi_A_^3+^] + *E^f^*[Bi_B_^3+^]}, yielding a value of −0.013 eV in YGG and −0.019 eV in YAG. The concentration of Bi^3+^ dimers, C_dimer_, is defined as $N_{\mathrm{site}}\left(C_{\mathrm{Bi}}\right)\left(C_{\mathrm{Bi}}\right)\;\text{×}\;\exp\left(-E_{\mathrm{binding}}/\mathrm{kT}\right)$, where k is the Boltzmann constant and T is the temperature. Hence, the ratio of C_dimer_ to C_Bi_ is 3.33 × C_Bi_ for YGG and 3.51 × C_Bi_ for YAG. At the same time, the concentration of Bi^3+^-doped garnets ranges from 1% to 10%, such as 5% as reported by Liu et al. and 7% as reported by Dong et al. [20,21]. Thus, the ratio of C_dimer_ to C_BiY_ is estimated to be approximately 0.1. Compared with the ratio of C_BiY_ to C_BiGa_, the Bi dimers are more readily formed and can generate the observable emission.

**Figure 6:**
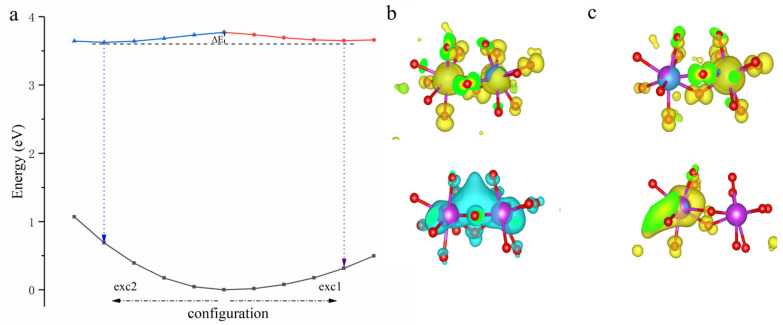
(**a**) The configuration coordinate diagram of the Bi^3+^ dimer in YGG. The exc1 and exc2 represent the relaxed path of the excited state of the Bi^3+^ dimer, and the ∆E is the energy barrier between the two excited states. (**b**,**c**) The charge densities of the excited state of exc1 and exc2, with the upper presenting the density of the hole and the lower presenting the density of the electron.

The inversion symmetry between the two nearest oxygen-sharing Y sites allows both the ES1 and ES2 excited states to form, as shown in Figure 1. The corresponding emission energies of the ES1 state and ES2 state are 3.1 eV and 2.8 eV in YGG, and 3.3 eV and 2.9 eV in YAG, as listed in Table 5. It should be noted that the orbital hybridization induced by the PBEsol function will overestimate the emission by about 0.3 eV, as reported by Chen et al. [48]; thus, the actual values are expected to be about 0.3 eV lower. To understand the stable excited states of Bi^3+^ dimers, the coordination configuration of Bi^3+^ dimers in YAG obtained via linear interpolation is drawn in Figure 6a. In the exc1 path, the Bi^3+^ dimers relax from ground state to the ES1 state, and in the exc2 path, the Bi^3+^ dimers relax from ground state to the ES2 state. It is interesting that the energy difference between the ES1 state and ES2 state at their equilibrium configuration is less than 0.1 eV. In Chen’s work, the IVCT state was a more stable state due to the Bi dimers in the LaOCl system exhibiting a center inversion symmetry [48]. In contrast, in the garnet systems, the Bi dimers exhibit mirror symmetry, where the 6p orbitals are not in a state of hybridization equality. In the charge density of the ES1 state (upper part of Figure 6b), the hole is localized at two Bi^3+^ ions unequally, as the hole density in the left Bi^3+^ is larger than in the right Bi^3+^. Meanwhile, the electron density of the right Bi3+ is larger than that of the left Bi^3+^, presenting a tendency consistent with the IVCT. Except for the smaller energy difference between the ES1 and ES2 states, the shallow energy barrier (∆E~0.15 eV) permits rapid relaxation between the two states at room temperature. Two visible emission bands can then be generated, which is consistent with the experimental results.

## 4. Conclusions

In this work, the ∆SCF methods were employed to clarify the origin of the complex emission of Bi^3+^ doped in YAG and YGG. Formation energy calculations confirm previous reports that Bi^3+^ ions predominantly occupy the Y site in both YAG and YGG crystals. The calculation of the excitation and emission processes of the isolated Bi^3+^ ion provides evidence that the experimentally observed violet emission can be assigned to the ^3^P_1_→^1^S_0_ transition of Bi^3+^ at Y sites. Furthermore, charge transition level (CTL) and energy barrier calculations reveal that the long afterglow emission of Bi^3+^ is associated with the trapping and detrapping of carriers at vacancy-related defects. To better understand the emission properties of Bi^3+^ doped garnets, the correlation between the luminescent properties and coordination environment, including the nephelauxetic effect and crystal field splitting, was discussed in the VRBE mode. Finally, the formation and transition processes of Bi^3+^ dimers were calculated. The calculated binding energy of Bi^3+^ dimers indicates that Bi^3+^ dimers can readily form at Y sites. Both the [6s3,6p1] and IVCT states can generate proper emission, due to the narrow energy barrier between the two excited states. The results provide insight for analyzing the influences of site occupation and understanding the relationship between the coordination environment and emission properties of Bi^3+^-doped garnets.

## Figures and Tables

**Table 3:** Experimental and calculated excitation and emission energies (in eV) of MMCT and the A band of Bi^3+^ in YAG and YGG.

		YGG			YAG		
		Bi^3+^_Y_	Bi^3+^_Ga1_	Bi^3+^_Ga2_	Bi^3+^_Y_	Bi^3+^_Ga1_	Bi^3+^_Ga2_
A band	ex	4.35	3.58	2.82	4.41	3.77	2.88
	em	4.08	3.47	2.71	4.22	3.64	2.67
MMCT	ex	4.32	3.55	3.46	5.24	4.42	4.35
	em	3.63	2.46	2.52	4.58	3.21	3.34
exp	ex	4.29 [20], 3.75 [21], 3.54 [21]			4.43 [16]		
	em	3.32 [21], 2.92 [21], 2.61 [21], 2.53 [21], 3.96 [20]			4.07 [16], 2.63 [16], 2.95 [17]		

**Table 4:** Average bond length (Å) between cation ions and oxygen ions in PBEsol optimization. Here, Bi^3+^* represents trivalent bismuth ions at the ^3^P_1_ state.

		Original	Bi^3+^-O	Bi^3+^*-O	Bi^4+^-O
YGG	Y	2.3782	2.4454	2.4906	2.3784
	Ga1	1.9967	2.2730	2.2721	2.1852
	Ga2	1.8510	2.1491	2.1570	2.0698
YAG	Y	2.3661	2.4328	2.4533	2.3704
	Al1	1.9239	2.2616	2.2602	2.1724
	Al2	1.7728	2.1392	2.1441	2.0602

**Table 5:** The binding energy, distance between two paired Bi^3+^ ions, and emission energy of Bi^2+^_Y_-Bi^4+^_Y_ (Em1) and Bi^2+^_Y_-Bi^4+^_Y_ (Em2). All results were based on the PBEsol function.

	Pair Distance (Å)	E_binding_ (eV)	Em1	Em2
YGG	3.787	−0.013	3.1	2.8
YAG	3.698	−0.019	3.3	2.9

**Figure A1:**
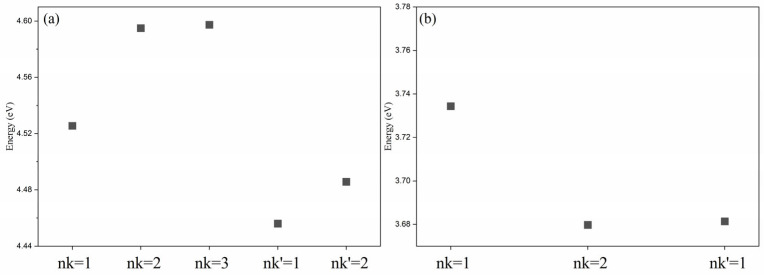
Convergence tests of k-grids and cell size were carried out for (**a**) ε(0/+1) of Bi_Y_^3+^ and (**b**) ^3^P_1_-^1^S_0_ transition of Bi_Y_^3+^ in a YAG host. For the 80-atom cell, k-grids are specified as nk × nk × nk, while for the 160-atom cell, they are denoted as nk′ × nk′ × nk′. All results were obtained using the PBEsol function. Thus, the value of ε(0/+1) and the ^3^P_1_-^1^S_0_ transition is different from the values obtained with HSE06.

**Figure A3:**
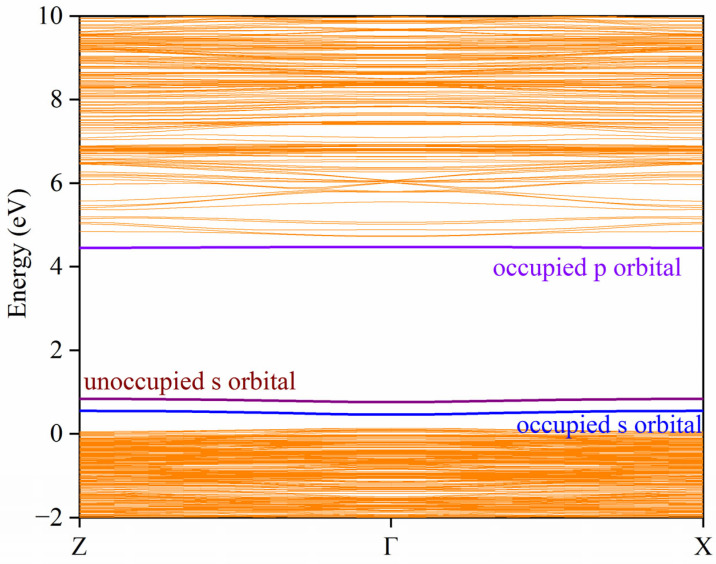
The band structure of Bi^3+^_Y_ in the YAG host. The K-PATH is different from the pristine cell, as the Bi-doping breaks the symmetry.

## Data Availability

The original contributions presented in this study are included in the article. Further inquiries can be directed to the corresponding author.

## Appendix A

Figure A1 shows the convergence test of k-grids and cell size. The YAG is chosen as the test platform. Figure A1a shows the results of ε(0/+1) of Bi^3+^ at Y sites. The energy difference between 1 × 1 × 1 grids and 4 × 4 × 4 grids is about 0.08 eV. At the same time, the energy difference between the 80-atom system and the 160-atom system is about −0.08 eV. Figure A1b shows the test results of the ^3^P_1_-^1^S_0_ transition of Bi^3+^ at Y sites. The energy difference between the 1 × 1 × 1 grids and 2 × 2 × 2 grids is less than 0.06 eV. And the energy difference between the 80-atom system and the 160-atom system is less than 0.06 eV as well.

Figure A3 shows the band structure of the ^3^P_1_ state of Bi_Y_ in YAG. The structural relaxation breaks the crystal symmetry, resulting in the splitting of degenerate bands. Both the s and p orbitals show flat band property.

Figure A4 presents the configuration coordinates of the main defects in the YAG host. Here, we consider the classical carrier trapping process, in which traps capture carriers from the band edges. The structures are generated by linear interpolation, and the results are calculated with the PBEsol function. All defects show a harmonic relation between the energy and configuration, except oxygen vacancy, which exhibits strong anharmonic relationships. V_Y_^3−^ defects present a classical energy barrier for trapping a hole, with a barrier of about 1.0 eV. At the same time, Y_Al_^0^ defects exhibit the lowest energy barrier with a value less than 0.05 eV, acting as a shallow electron trap. V_Al1_^3−^ and V_Al2_^3−^ defects show a larger energy barrier for hole trapping, with values greater than 1.0 eV. Oxygen vacancies exhibit a larger classical energy barrier as an electron trap, with a value greater than 2.0 eV. For these three types of defects, the tunneling effects should be important, as discussed in Alkauskas’s work [49].
